# Chromosome Instability in Fanconi Anemia: From Breaks to Phenotypic Consequences

**DOI:** 10.3390/genes11121528

**Published:** 2020-12-21

**Authors:** Benilde García-de-Teresa, Alfredo Rodríguez, Sara Frias

**Affiliations:** 1 Laboratorio de Citogenética, Instituto Nacional de Pediatría, Ciudad de México 04530, Mexico; b.garciadeteresa@gmail.com; 2 Doctorado en Ciencias Biomédicas, Universidad Nacional Autónoma de México, Ciudad de México 04510, Mexico; 3 Instituto de Investigaciones Biomédicas, Universidad Nacional Autónoma de México, Ciudad de México 04510, Mexico

**Keywords:** chromosomal instability, FA pathway, radial figures, TGFβ pathway, MYC, p53, bone marrow failure, cancer, physical abnormalities, infertility

## Abstract

Fanconi anemia (FA), a chromosomal instability syndrome, is caused by inherited pathogenic variants in any of 22 *FANC* genes, which cooperate in the FA/BRCA pathway. This pathway regulates the repair of DNA interstrand crosslinks (ICLs) through homologous recombination. In FA proper repair of ICLs is impaired and accumulation of toxic DNA double strand breaks occurs. To repair this type of DNA damage, FA cells activate alternative error-prone DNA repair pathways, which may lead to the formation of gross structural chromosome aberrations of which radial figures are the hallmark of FA, and their segregation during cell division are the origin of subsequent aberrations such as translocations, dicentrics and acentric fragments. The deficiency in DNA repair has pleiotropic consequences in the phenotype of patients with FA, including developmental alterations, bone marrow failure and an extreme risk to develop cancer. The mechanisms leading to the physical abnormalities during embryonic development have not been clearly elucidated, however FA has features of premature aging with chronic inflammation mediated by pro-inflammatory cytokines, which results in tissue attrition, selection of malignant clones and cancer onset. Moreover, chromosomal instability and cell death are not exclusive of the somatic compartment, they also affect germinal cells, as evidenced by the infertility observed in patients with FA.

## 1. Introduction

Fanconi anemia (FA) is a rare disease with an incidence of 1–5 per million of births and is the most commonly inherited bone marrow failure syndrome [1]. FA is caused by the failure of the Fanconi anemia/breast cancer (FA/BRCA) pathway [2]; thus far, 22 genes (*FANCA* to *FANCW*) that participate in this pathway have been identified. Germline pathogenic variants (PV) in any one of these genes are the origin of this disease [3]. PV show an autosomal recessive inheritance for 20 of these genes, autosomal dominant inheritance has been shown for one gene (*FANCR/RAD51*) and X-linked inheritance for another gene (*FANCB*). Inherited PV in the *FANCA*, *FANCC* or *FANCG* genes account for approximately 90% of FA cases, whereas the other 19 *FANC* genes account for the remaining 10% [3]. The FA/BRCA pathway is involved in the proper functioning of various cellular processes; one of its most important functions is during the repair of DNA interstrand crosslink (ICL), lesions that covalently join the two DNA strands and impair DNA replication and transcription. In everyday life, we are exposed to sources of ICL-inducing agents that can be both endogenous resulting from cellular metabolism or exogenous due to environmental, occupational or personal exposure habits. Some calculations suggest that in the steady state every cell could carry around 37,000 lesions per genome, and that ICLs account for approximately 1500 of these lesions, whose origin can be bi-functional chemicals related to formaldehyde, acetaldehyde, acrolein and in smaller proportions crotonaldehyde [4]. ICLs of exogenous origins are more difficult to assess since for all the agents with the capacity to induce them, only a small fraction (typically 1–5%) will be ICLs, while the majority of the induced DNA damage will be monoadducts or intrastrand crosslinks [5]. Most of this damage is successfully repaired via the FA/BRCA pathway.

Failure of the FA/BRCA pathway has consequences at various levels of complexity: (1) at the chromosomal level by the presence of numerical and structural chromosomal instability; (2) at the cellular level resulting in increased cell death, alteration of the cell cycle, high sensitivity to oxidative damage and to DNA cross-linking agents, both exogenous such as chemotherapeutic drugs, e.g., cis-platinum, mitomycin C or diepoxybutane, as well as endogenous aldehydes, a product of cell metabolic activities [6,7]; and (3) at the clinical level, where patients with FA present three main features: developmental abnormalities, bone marrow failure and an increased risk of cancer [8].

In this review, we present current knowledge on the function of the FA/BRCA pathway and discuss the negative consequences that result from the failure of this critical pathway at both cellular and organismal levels. We present mechanisms responsible for chromosome aberrations, examine the cellular response to DNA damage and discuss possible pathophysiological processes involved in the clinical consequences of DNA damage accumulation.

## 2. Double Strand Breaks Are at the Center of Chromosome Aberrations in FA

### 2.1. Involvement of FA/BRCA Pathway in DNA Repair

The protein products of the *FANC* genes collaborate in the FA/BRCA pathway to protect DNA replication fork and repair ICLs [1]. ICLs are dangerous lesions that prevent the opening of the double stranded DNA for transcription and replication. For ICLs repair, the cell needs to use several DNA repair converging mechanisms, and the FA/BRCA pathway is tasked with coordinating them to process the ICL into byproduct lesions, remarkably a DSB which is to be preferentially repaired in an error-free way. Individual functions of FANC proteins during this assemblage appear in Table 1.

**Figure 1:**
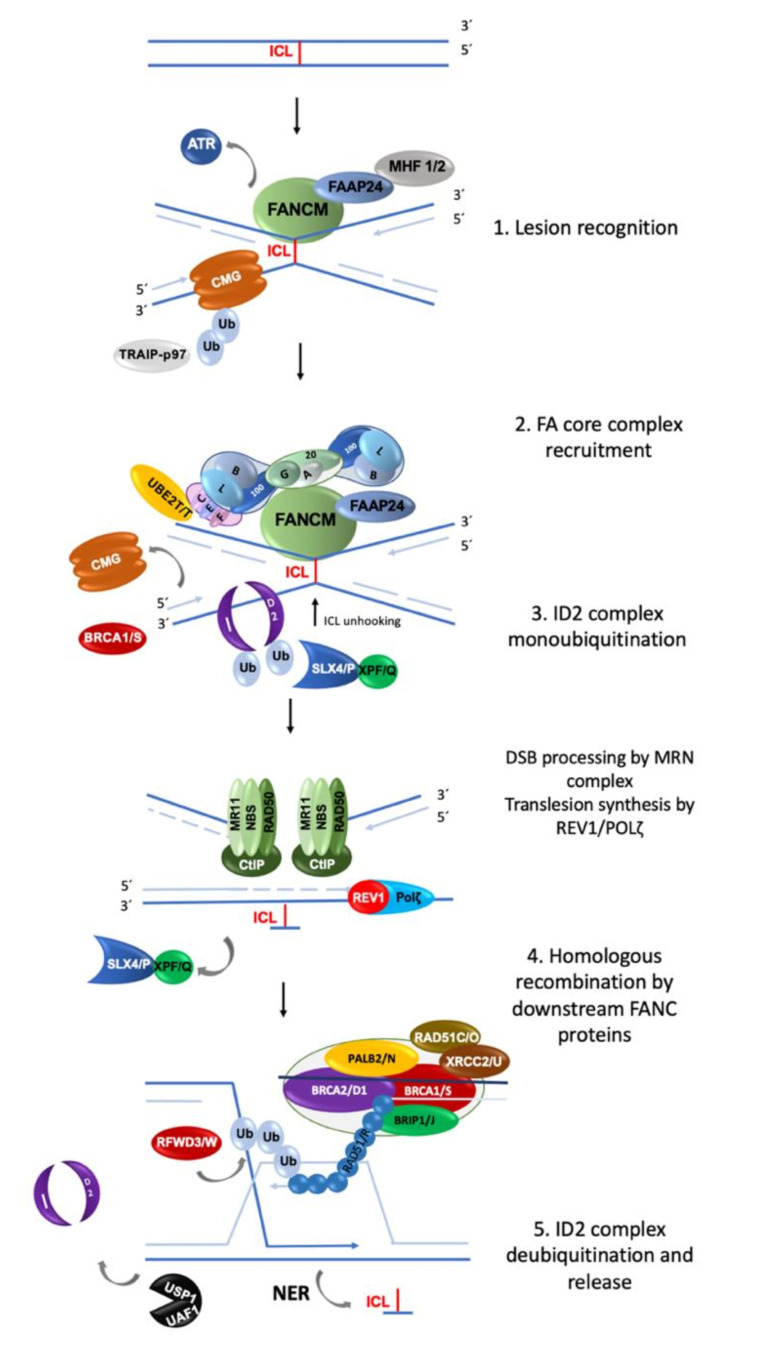
Summary of the activity of FANC proteins in the FA/BRCA pathway. The main function of this pathway is the removal of DNA interstrand crosslink (ICL); 22 FANC proteins participate in: (1) Lesion recognition. FANCM and its partners recognize ICLs during the convergence of two replication forks and promote ATR activation; the CMG helicase complex is unloaded to allow the approach of the leading strands to the ICL. (2) FA core complex recruitment. FANCM and its partners recruit the FA core complex and UBE2T/FANCT (the “upstream” proteins), to exert their E3-ubiquitin ligase activity and monoubiquitinate the FANCI and FANCD2 heterodimer (also known as the “ID2 or central complex”). (3) ID2 complex monoubiquitination. The monoubiquitinated central complex activates the endonucleolytic function of FANCP-SLX4-FANCQ/XPF resulting in the unhooking of the ICL from one of the DNA strands and the generation of a DSB. Both DNA ends of the DSB are processed by the MRN/CtIP complex to form a 3′ overhang. In the opposite strand, the unhooked ICL has now become an adduct; to bypass it, the REV1-polymerase ζ complex (including FANCV protein) performs translesion synthesis of the new strand. (4) The processed DSB is repaired by homologous recombination. The “downstream” FANCD1/BRCA2, FANCN/PALB2, FANCS/BRCA1, FANCJ/BRIP1, FANCO/RAD51C and FANCR/RAD51 proteins coordinate to coat the processed DNA strand of the DSB with RAD51/FANCR and paralogs RAD51C/FANCO, to invade the newly polymerase ζ-synthesized double strand of its sister chromatid, using it as a template to recover the original nucleotide sequence. (5) The cycle finishes with deubiquitination and unloading of the ID2 complex by USP1-UAF1 and the removal of the ICL-adduct by NER (nucleotide excision repair) pathway [9,12,22,23,24].

The FA/BRCA pathway is activated for repairing ICLs during the S phase of the cell cycle, when the replisome finds an ICL and two convergent replication forks become stalled [11]. The ICL repair process can be divided into modules of activity of the FA/BRCA pathway [12] (Figure 1).

(1) Lesion recognition. FANCM and its interacting partners, FAAP24, MHF1 and MHF2 [3], detect the lesion on the DNA when two replication forks converge at the vicinity of an ICL [11]. Replisome complexes are unloaded, leaving stalled replication forks with single stranded DNA (ssDNA) regions covered by Replication Protein A (RPA). This leads to an ATR/CHK1 signaling activation to trigger DNA damage checkpoints [13]. The best described scenario for triggering ICL repair implicates the convergence of two replication forks at the ICL site [11], the leading strand on one side of the ICL stops 20–40 nucleotides before the ICL, and then the CMG helicase is removed from the stalled fork, aided by the ubiquitin E3 ligase TRAIP, the p97 ATPase and FANCS/BRCA1 protein; the fork advances to nucleotide 1 with respect to the ICL and waits for the opposite fork to reach the ICL in a similar manner. Once FANCM and its interacting partners are in close proximity to the ICL, their key function is to recruit the members of the next module, the FA core complex, to the chromatin [14].

(2) FA core complex recruitment. The best described function of the FA core complex is as an E3-ubiquitin ligase that is integrated by the proteins FANCA, FANCB, FANCC, FANCE, FANCF, FANCG, FANCL, FAAP100, FAAP20, FAAP24 and FANCT. FANCL has the E3 ubiquitin ligase catalytic activity and FANCT bears the E2 catalytic activity. Three subcomplexes can be recognized: (a) the FANCB–FANCL–FAAP100 (BL100) subcomplex is important for the integration of all components of the FA core complex; (b) the FANCA–FANCG–FAAP20 (AG20) subcomplex is important for the nuclear localization of the entire multimer; and (c) the FANCC–FANCE–FANCF (CEF) subcomplex is in charge of bridging the FA core complex with the members of the module 3 (its target), the FANCI-FANCD2 (ID2) complex [3]. Once assembled, the entire FA core complex exerts its E3-ubiquitin ligase activity to add a ubiquitin group to FANCI at lysine 523 and to FANCD2 at lysine 561. 

(3) ID2 complex monoubiquitination. The FANCD2-FANCI heterodimer, frequently called the central complex, is recruited to the stalled fork, where FANCI is tri-phosphorylated by the ATR-kinase [15], stimulating the FA core complex mediated monoubiquitination of both FANCI and FANCD2. The tri-phosphorylation of FANCI also inhibits the deubiquitinase activity of the USP1-UAF1 complex over the ID2 complex until ICL repair and replication are completed [15]. The ubiquitinated ID2 complex protects the replication forks and regulates the activity of the proteins involved in the processing of the ICL, enabling the recruitment of the proteins of the fourth module.

(4) Homologous recombination (HR) by downstream proteins. Proteins acting downstream in the FA/BRCA pathway include BRCA2/FANCD1, BRIP1/FANCJ, PALB2/FANCN, RAD51C/FANCO, RAD51/FANCR, BRCA1/FANCS, XRCC2/FANCU, XPF/FANCQ, SLX4/FANCP, REV7/FANCV and RFWD3/FANCW, all of which are committed to remove the ICL and maintain genomic integrity through various types of DNA repair. The ubiquitinated ID2 complex recruits the FANCP/SLX4 scaffold protein, which in turn coordinates the endonucleolytic activity of FANCQ/XPF. This endonuclease makes DNA incisions on both sides of the ICL and unhooks it [16]. After ICL unhooking, different types of lesions are generated: one of the chromatids is left with a single strand DNA region with the unhooked ICL, and in the sister chromatid a double strand break (DSB) is generated (Figure 1). All these lesions are repaired by different DNA repair pathways that act coordinately in the FA/BRCA pathway.

The single strand region is repaired by translesion synthesis (TLS), through polymerases REV1 and the polymerase ζ complex (REV7/FANCV-REV3), an error-prone polymerase that uses as template the complementary strand with the adduct; while this allows the replication progress, the low fidelity of this polymerase can introduce errors in the nucleotide sequence [17]. This polymerase is necessary for ICL repair as part of the FA/BRCA pathway; indeed, its recruitment to ICL repair intermediates is performed by ubiquitinated PCNA and FA core complex [18]. The unhooked adduct in the opposite strand is repaired by nucleotide excision repair (NER); FANCQ/XPF protein participates in both FA/BRCA and NER pathways, however FA patients with mutations in this gene, do not share the phenotype with Xeroderma Pigmentosum patients, making evident that is a multitask protein [19]. During ICL repair, FANCQ/XPF makes incisions around the ICL and NER polymerase eta (POL η) is recruited by FANCD2. FA proteins FANCM and FANCT have been implicated in the regulation of NER; these data show the crosstalk between FA/BRCA and NER pathways in the ICL repair [9].

The ICL-associated DSB is processed by the proteins downstream of the FA/BRCA pathway (FANCD1/BRCA2, FANCN/PALB2, FANCS/BRCA1, FANCJ/BRIP1, FANCO/RAD51C and FANCR/RAD51); this set of proteins is recruited by ubiquitinated ID2 complex and performs an homology-directed repair, using the recently restored sister chromatid to perform the error-free HR repair. When the lesion is repaired, the cell is able to continue the cell cycle. Interestingly, some of the downstream proteins have also recently been shown to have functions upstream of the ubiquitinated FANCI-FANCD2 complex. For example, deubiquitinated FANCI and FANCS/BRCA1 are involved in the recruitment of the FA core complex [20]; FANCS/BRCA1 is required for positioning FANCD2 at the ICL site, whereas FANCD1/BRCA2 and FANCJ promote the FANCD2 chromatin localization [9]. Importantly, individuals who are heterozygous for PV in genes of Module 4 are at high risk for developing breast and ovarian cancer [2,21].

(5) ID2 complex deubiquitination. After ICLs repair the activity of the replication fork is restarted; the last module includes the deubiquitination of FANCD2/FANCI activated complex, leading to the re-start of the DNA synthesis by the canonical DNA polymerases. The deubiquitination is performed by the USP1-UAF1 complex resulting in the release of the ID2 complex from the chromatin to complete the ICL repair cycle [12].

### 2.2. Repair of Double Strand Breaks

DSBs are generated as a byproduct during the processing of ICLs by the FA/BRCA pathway. DSBs are considered one of the most toxic lesions for cells; misrepair may originate mutations and chromosomal abnormalities that may lead to cell death or tumorigenesis, therefore the accurate repair of this type of lesion is essential to maintain genomic stability and cell viability.

Several factors influence the processing and repair of DSBs including the phase of the cell cycle in which the damage occurs, their origin (associated to replication fork stalling or replication-independent) and the number of DSB events in the same cell, among others. The two major repair mechanisms for DSBs are HR and non-homologous end joining (NHEJ); for the latter, a canonical NHEJ (cNHEJ), and an alternative pathway, also called microhomology-mediated end joining (MMEJ), have been described (Table 2). HR uses an intact homologous sequence as a template for the repair of DSBs. For this reason, HR is carried out during the post-replicative period of the cell cycle, which includes the S and G2 phases, when a sister chromatid is available; the free DNA ends have to search for the homologous sequences, thus requiring extensive DNA resection and processing. On the contrary, the ligation of non-homologous ends performed by NHEJ requires minimal or no sequence homology and allows ligation of DNA ends with minimal processing.

In normal cells, NHEJ efficiently joins the correct DNA ends of a DSBs, without the formation of chromosomal aberrations, although the original DNA sequences flanking the DSB may not be exactly restored, due to small losses of nucleotides that occur during the DNA end-processing that is needed for successful NHEJ. However, if multiple DSBs occur simultaneously, the activity of NHEJ, which can be independent of template and homology, may lead to ligation of wrong DNA ends generating gross chromosomal rearrangements [22].

#### 2.2.1. Homologous Recombination

**Figure 2:**
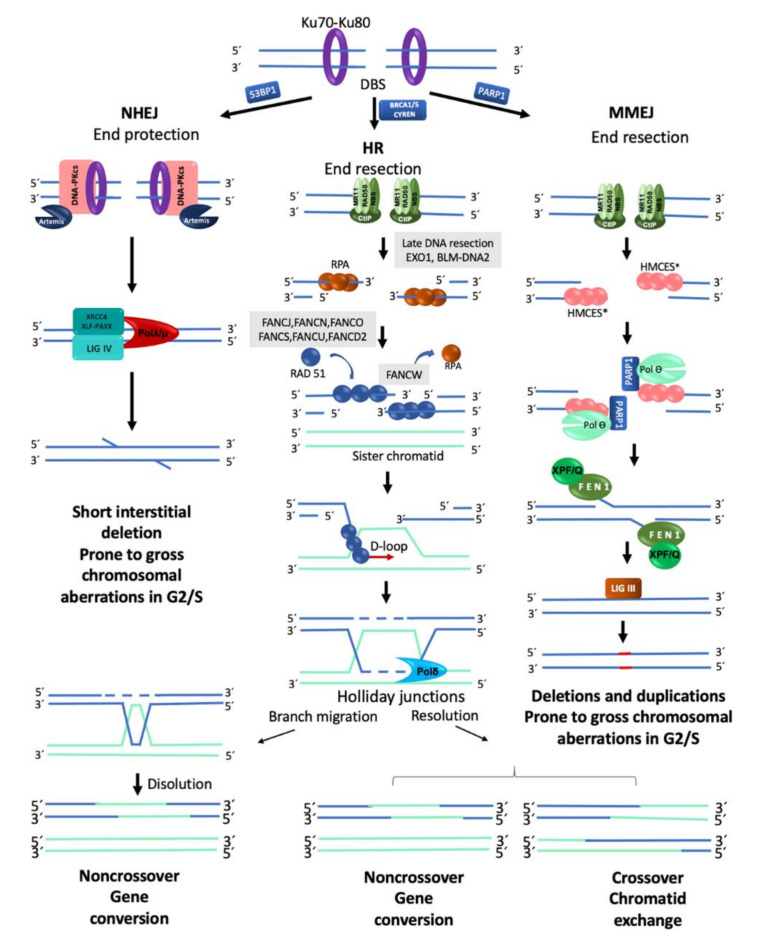
Major mechanisms of double-strand break repair. NHEJ, Non-Homologous End Joining. DNA ends are protected by KU70/KU80, which prevents their end-processing. When the ends are incompatible, a segment of up to four nucleotides is detected, Artemis eliminates the remaining incompatible segments and ligation is carried out by LIGIV-XRCC4. This pathway can repair a DSB without chromosome modification; however, during S/G2 and in the presence of several DSBs, it is considered prone to generate chromosomal alterations. HR, Homologous Recombination. This pathway is only available during the S/G2 phases of the cell cycle since it requires a homologous template; HR is the best choice to maintain sequence fidelity because in general it repairs in an error-free manner. A ssDNA 3′ overhang is produced by the action of the MRN-CtIP complex; it is first covered by RPA proteins which are later replaced by RAD51 to form the nucleoprotein strand that will carry out the invasion of the DNA of the sister chromatid in order to use it as a template to restore the continuity of the original nucleotide sequence. MMEJ, Microhomology-Mediated End Joining. PARP1 prevents KU70/KU80 binding to DNA ends and allows the recruitment of MRN-CtIP to initiate end-resection, creating a short 3′ overhang. This overhang is preferentially covered by HMCES which channels the damage to be repaired by MMEJ instead of HR. PARP1-POLQ search for microhomology of 2–20 bp and align the strands. The resulting flaps are eliminated by XPF/FANCQ-FEN1. Alternatively, POLQ can direct DNA synthesis to add nucleotides to make DNA ends compatible; this end processing generates *in situ* deletions and duplications. In addition, if this pathway is active during S/G2, and several DSBs coincide in time and space, gross chromosomal aberrations are formed because, similar to NHEJ, they do not require long stretches of homology to ligate the DNA ends [23,26]. * RPA or HMCES (5-hydroxymethylcytosine binding, embryonic stem cell-specific protein) [26,28].

In a normal cell, the FA/BRCA pathway continues “downstream” after the generation of a DSB, using the HR repair pathway to join the DNA ends in an error free manner. HR is restricted to the S and G2 phases of the cell cycle, using the sister chromatid as template to recover the original nucleotide sequence [22]. However, in any phase of the cell cycle, Ku70-Ku80 are abundant proteins that bind the broken DNA ends and protect them [25]. Since DSBs in S/G2 phases are preferentially repaired by HR, the Ku70-Ku80 heterodimer is removed by proteins that process the DNA ends. A first step in this processing is mediated by the MRN complex (MRE11–RAD50–NBS1), which, aided by CtIP (CtBP-interacting protein), introduces an endonucleolytic nick up to 300 bp away of the DSB site [22]. Next, the 3′ to 5′ MRN exonuclease activity extends the nick forming a 3′ overhang. This process finally ends up displacing the Ku70-Ku80 proteins and elicits the entrance of the late DNA end resection proteins EXO1 (exonuclease1) and BLM-DNA2 (bloom syndrome helicase-endonuclease2) [22,27]. These proteins facilitate unwinding of the DNA and digestion of the 5′ strand to lengthen the 3′ overhang, which allows the entry of the RPA protein complex, to protect the single stranded DNA and proceed to HR repair by the FANC proteins (Figure 2) [22].

**Figure 3:**
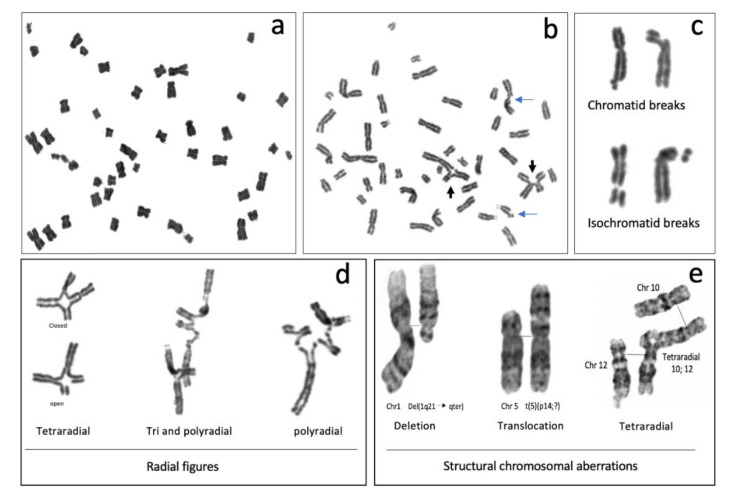
Representative metaphases and common structural chromosome aberrations observed in FA cells cultured with 0.1 µg/mL DEB: (**a**–**d**) peripheral blood lymphocytes from a patient with FA and (**e**) from a patient derived, *FANCA* mutated VU817 lymphoblastoid cell line. (**a**) Endoreduplication. (**b**) Metaphase with structural chromosomal aberrations; blue arrows show chromatid breaks and black arrows show radial figures. (**c**) Breaks. The images show how the broken fragments are kept very close to the chromosome that originated them because there is a cohesion of chromatids in metaphase. (**d**) Radial figures. In the first column can be observed that the tetraradial can be closed when the four DNA ends of the two non-homologous chromosomes are rejoined or open when only two of the four DNA ends were rejoined. (**e**) GTG banded chromosomes reveal other gross structural chromosomal aberrations that may be found in FA cells, such as deletions and translocations. In the radial figure, normal chromosomes 10 and 12 are aligned with the tetraradial, to highlight the trajectory of the rearrangement.

The mediators of HR, FANCD1/BRCA2, FANCS/BRCA1, FANCN/PALB2 and FANCJ/BRIP1, act by displacing RPA from the single stranded DNA and loading FANCR/RAD51 and its paralogs FANCO/RAD51C and FANCU/XRCC2 into the ssDNA, which leads to the formation of a nucleoprotein filament with the capacity to invade the sister chromatid and search for homologous sequences that will be used as template to restore the original sequence that was interrupted by the DSB. This nucleofilament assists the base-pairing when the complementary sequences in the sister chromatid have been found (synapsis). Additional FANC proteins mediate the homology search, including FANCW/RFWD3, a ubiquitin E3 ligase that regulates the turnover of RPA by FANCR/RAD51, initially promoted by FANCS/BRCA1. Interestingly, FANCS/BRCA1 has also been shown to promote DNA end resection, RAD51 loading and collaborate in the homology search mediated by FANCR/RAD51, highlighting the multiple roles of FANCS/BRCA1 [22,27] (Figure 2 and Figure 3).

When the synapsis has been stabilized, dissociation of FANCR/RAD51 is required to promote DNA synthesis. A displacement-loop (D-loop) is then formed allowing the engagement of the DNA polymerase δ (Pol δ) [14,23,26,29] to incorporate nucleotides and synthesize new DNA. The DNA heteroduplex leads to the formation of Holliday junctions, which are resolved by helicases and endonucleases. The Holliday junctions can be either dissolved or resolved, the first option restores the original sequence by gene conversion (non-crossover), whereas the second can promote sister chromatid exchanges (crossover) or gene conversion (non-crossover), this prevents chromosomal translocations, as expected by an error-free repair [30,31] (Figure 2). Importantly, HR preferentially uses the sister chromatid as a template due to its perfect homology and close proximity, though the use of the homologous chromosome is also possible, however this alternative is less efficient and can generate regions of homozygosity in the next cell generation.

Once the DSB is repaired, FANCD2-Ub has to be extracted from the lesion; for this to occur, it has to be deubiquitinated by the USP1-UAF1 deubiquitinase complex and p97. This deubiquitination step is the process best-known to contribute to finalize the HR [12,32], however all known “downstream” FANC proteins not only have important functions in HR, but some of them also control the start and conclusion of the repair cycle, as well as the DSB repair pathway choice [26].

During the processing of DNA ends, the ATM (ataxia telangiectasia mutated) and ATR (ataxia telangiectasia related) kinases become activated by MRN and RPA, respectively, and regulate additional aspects of the cell’s DNA damage response, including activation of the cell cycle checkpoints.

In patients with FA, the error-free FA/BRCA pathway is not functional, so their cells have to select an alternative error-prone pathway to repair their DNA. The routes they usually use are the NHEJ and the alternative end joining also called microhomology mediated end joining (MMEJ). These error prone pathways are described below.

#### 2.2.2. Non-Homologous End Joining

NHEJ is the dominant pathway for the repair of DSBs in the human cells [25]. Processing of a DSB by NHEJ is notably different from HR. As observed in Table 2, the abundance and availability of its components throughout all the cell cycle and the speed of DSB repair kinetics (15–30 min) explain the dominant role that cNHEJ has in the preservation of genome integrity. The initiation of the classical cNHEJ requires the union of the Ku70/Ku80 heterodimer to the broken DNA ends. This protects the DNA from the exonuclease activity of proteins such as MRN or EXO1.

Ku70/Ku80 is also a platform for the recruitment of other DNA repair proteins, such as DNA-dependent protein kinase catalytic subunit (DNA-PKcs) and its cofactor with exonuclease activity called Artemis. The Ku70/Ku80-DNA-PKcs complex makes a first synapse between the two DNA ends followed by a second synapse with a closer contact operated by the proteins DNA ligase IV (LIG4), XRCC4/XLF, PAXX, DNA polymerases λ and μ, Aprataxin and PNK-like factor (APLF). These proteins are also in charge of performing DNA end processing by removing some nucleotides from the broken ends to allow the ligation between either blunt DNA ends or DNA ends with a very short resection that leads to small single stranded DNA overhangs (Figure 2). This resection (≤4 pb), although small, can change the information in the damaged site, explaining part of the errors associated with DNA repair by cNHEJ [22,25,26]. The cNHEJ simplicity explains in part its high speed, enabling the re-ligation of DSBs shortly after they were formed. High speed in cNHEJ partially compensates for the lack of homology use. When a single DSB occurs, the proximity and topology of the two original DNA ends increases the probability of its re-ligation; however, when more than two DNA ends coexist, the template-independent ligation that characterizes NHEJ increases the probability of generating gross chromosomal rearrangements [33].

#### 2.2.3. Alternative End Joining (Microhomology Mediated End Joining)

When HR or cNHEJ are unavailable, the cell can use MMEJ; the distinctive characteristic of this repair pathway is the use of very short homologous sequences (2–20 bp) to elicit the re-joining of the two DNA ends. MMEJ is an error prone pathway with characteristics similar to the cNHEJ but that also includes DNA end processing. MMEJ requires PARP1 (poly(ADP-ribose) polymerase 1), a protein that competes with KU for the DNA ends generated by a break [34], the binding of PARP1 facilitates the recruitment of DNA polymerase θ (Pol θ) to DSBs [35]. For DNA end resection, MMEJ recurs to CtIP and the MRN complex to create a 15–100-nucleotide 3′ overhang. This ssDNA 3′ overhang coated by proteins RPA or HMCES (5-hydroxymethylcytosine binding, embryonic stem cell-specific protein) [28] makes a short displacement until it finds a microhomology region of up to 20 bp, leaving a 5′ flap. At this point, HMCES are unloaded through the Pol θ-associated helicase activity, the displaced 5′ ssDNA flaps are removed by FEN1 (flap endonuclease 1) and DNA ligase I or DNA ligase III closes the dent [36] (Figure 2).

When no homology is found, the polymerase activity of Pol θ is turned on to add nucleotides and provide the necessary microhomology to stabilize the junction between the two free DNA ends; either because it removes nucleotides to match existing microhomology regions in DNA ends or because it inserts nucleotides to create microhomology regions, MMEJ is prone to introduce deletions and duplications [26,28]. Pol θ is enhanced in HR defective cells, suggesting that this type of repair may act when the DNA ends cannot be repaired by the cNHEJ [26]. The high levels of chromosome translocations observed in cNHEJ mutants that use MMEJ indicate that this process tends to bind together non-homologous segments and therefore produces structural chromosomal aberrations (SCA) [28,36]. Apparently, MMEJ functions as a backup route when HR and cNHEJ fail to resolve the DSBs present in the cell. [33].

#### 2.2.4. DSB Repair Pathway Choice

Some calculations suggest that the cell responds to even a single DSB by activating cell cycle checkpoints; it has been estimated that the integrity of the genome and cell survival is put at risk when several DSBs (~10) are simultaneously induced [37]. Therefore, the choice of the DNA repair pathway to maximize the efficiency to preserve genome integrity is critical for the survival of any cell. Although several pathways and sub-pathways have been implicated in the repair of DSBs, here we only consider the three main pathways: HR, NHEJ and MMEJ.

The phase of the cell cycle in which a DSB occurs is one of the most important and defining characteristics for DNA repair pathway choice. HR repair is not active in G1 because sister chromatids are not available, therefore DSBs appearing in this phase will be channeled to NHEJ or MMEJ. When a DSB occurs in S/G2 phases, HR is the preferred pathway for its repair since it is the best way to preserve the integrity of the DNA sequence, even if end joining mechanisms are active. Although the exact mechanism behind the DSB repair pathway choice remains elusive, proteins driving the initial steps of DSB processing are the candidates to determine the selection of the best pathway [26].

Nucleolytic processing of the DNA-ends is a critical step during DSB repair pathway choice (Figure 2). During the G1 phase of the cell cycle, an active suppression over end resection machinery, specifically MRN, is performed by the 53BP1 protein and the shieldin complex, thus restricting HR to S/G2 phases, and leaving the Ku70/Ku80 heterodimer without competitors during its DNA end-protection activity. Through suppression of DNA end processing, 53BP1 favors NHEJ. During the postreplicative phases (S and G2), the FANCS/BRCA1 protein, in collaboration with MRN and CtIP, antagonizes 53BP1 and promotes the essential DNA end resection step for both HR and MMEJ [38]. Posttranslational modifications (PTM) of histones have been shown to be important for chromatin localization of 53BP1: it requires the combined interaction of its Tudor domain to H4K20me2 and the H2AK15ub through a C-terminal ubiquitin-interacting motif [39]. The FA/BRCA pathway, via FANCD2, restrains the accumulation of 53BP1 by regulating the activity of TIP60, an acetylase of histone H4 that increases the presence of H4K16ac and H2AK15ac in the site of DNA damage hindering access to the post-translational modification required to maintain 53BP1 in the chromatin. Failure of the FA/BRCA pathway leads to 53BP1 accumulation favoring NHEJ, leading to chromosomal aberrations [12].

The HR, NHEJ and MMEJ pathways are all active during S/G2; therefore, to channel DSBs repair to HR during this cell cycle phase, it is necessary to repress the activity of cNHEJ. Recently, a specific inhibitor of NHEJ in the post replicative phase has been proposed, CYREN (cell cycle regulation of NHEJ), which binds the Ku70/Ku80 complex and regulates the DNA repair pathway choice by inhibiting NHEJ and promoting HR when a sister chromatid is available to allow error free recombination [40].

### 2.3. Double Strand Breaks as the Substrate for Chromosomal Aberrations

The most common CAs observed in metaphase spreads from patients with FA are chromatid or isochromatid breaks, deletions, duplications, fragments and gross chromosomal aberrations, such as translocations, dicentric chromosomes, radial figures and other complex rearrangements (Figure 3) [41]. The formation of all of these SCAs involves breaking and rejoining of DNA molecules, therefore DSBs are considered to be the origin of SCA [42].

The heterogeneous clinical phenotype observed in the patients with FA contrasts with their highly constant cellular and cytogenetic phenotype. The homogeneous cellular phenotype observed in FA indicates that failure in any stage of the FA/BRCA pathway results in the incapacity for repairing ICLs and DSBs in an error-free manner. Misrepaired DSBs in particular, which arise after initial ICL processing, are the main source of chromosomal aberrations (CA) present in FA cells, and this sensitivity has been critical for the diagnosis of FA, which is largely based on the detection of CA observed in cell cultures treated with Diepoxybutane (DEB) and mitomycin C (MMC) [43].

#### 2.3.1. Non-Rejoined Structural Chromosomal Aberrations: Breaks

In FA cells, chromosome breakage is the result of initiated but unfinished ICL repair. Most of the breaks in FA cells are of the chromatid type (Figure 3b,c), indicating that they were formed during the post-replicative period of the cell cycle, and therefore only one chromatid is affected, even though the chromosome is already composed by two sister chromatids. Generally, when a break of the chromosomal type (both chromatids are broken) is detected, it can be inferred that a DSB occurring during the G1 phase is the cause. Nonetheless, the FA pathway operates in the S/G2 phases, therefore it is more likely that chromosomal breaks are the result of two very close DSBs, one in each chromatid, and can be considered isochromatid breaks. Isochromatid breaks are less common than chromatid breaks when metaphase spreads from FA samples are analyzed.

Since the FA/BRCA pathway is not functional in FA cells, the presence of chromosome breaks, when FA cells are treated with ICL inducing agents, suggests that endonucleases, alternative to the canonical FA/BRCA pathway, unhook the ICL and generate a DSB. This DSB however is not channeled to HR by the downstream modules of the FA/BRCA pathway and might remain unrepaired; when a cell reaches metaphase, the sites of these unrepaired DSBs can be visualized as chromatid breaks. Of note, the piece of broken chromatid usually remains adjacent to its chromosome due to the mitotic chromatin structure and the cohesin proteins that hold together the sister chromatids and prevent their separation until anaphase [44] (Figure 3c).

#### 2.3.2. Rejoined Structural Chromosome Aberrations

In FA cells, the presence of translocations, dicentrics and radial figures makes evident the relevance of FA/BRCA pathway in the protection against SCAs, since all of these aberrations originate by ligation of two or multiple broken DNA ends with little or absent homology. If the end joining pathways cNHEJ and MMEJ operate during S/G2 phases of the cell cycle, when the replicated chromosome is composed by two sister chromatids, the joining of one of these chromatids with a non-sister chromatid from a different chromosome (homologous or non-homologous) will lead to SCA formation (Figure 3).

Radial figures are formed when at least two DSBs from non-sister chromatids are joined together. In these two DSBs, four DNA ends are available; therefore, if the four DNA ends are joined by an error prone DNA repair machinery, a closed tetraradial can be generated. However, an open tetraradial figure will be originated if only two DNA ends are rejoined (Figure 3d). A triradial figure has the pre-requisite of three DSBs, one of them in a chromatid of the receptor chromosome and two more (of the isochromatid break type), in the second chromosome to join one chromatid of the receptor chromosome; in this way, polyradial figures require several DSBs for their formation (Figure 3d). In FA cells, radial figures form between non-homologous chromosomes (Figure 3e) [45], both spontaneously and induced by MMC or DEB. When proteins in both the HR and cNHEJ pathway are inactivated, an increase in the frequency of radial figures can be observed [46], suggesting that the MMEJ pathway highly contributes to its generation.

**Figure 4:**
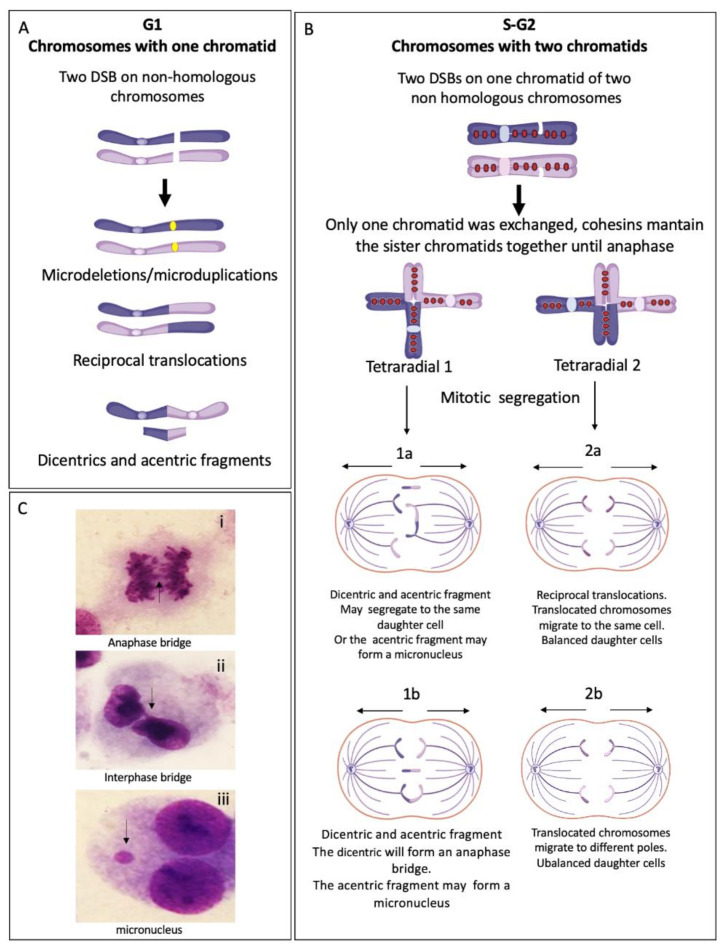
End joining repair pathways outcomes when more than one DSB is present in the same space-time and the rejoining is between non-homologous chromosomes. (**A**) G1 chromosomes have only one chromatid. When two chromosomes have DSBs, repair by NHEJ and MMEJ can perform the reunion of the two original chromosomal fragments, without error or leaving microdeletions or microduplications. If two fragments from different chromosomes are joined, translocations or dicentrics + acentric fragments are generated. (**B**) S/G2 chromosomes have two sister chromatids linking each other by cohesins. If only one sister chromatid has a DSB, the interchange of segments during repair generates gross structural aberrations such as translocations or radial figures, which may have several configurations depending on the rejoined fragments. Here, we show two possible tetraradial figures with different outcomes after segregation. (1) The segregation of a closed tetraradial with two DNA ends rejoining chromatids with centromere: a dicentric. (1a) In this type of segregation, both normal chromosomes segregate in a daughter cell and the dicentric chromosome moves together with the acentric fragment to the second daughter cell. (1b) The normal chromosomes segregate each to a different daughter cell, the dicentric is attached to both centrosomes of the mitotic spindle, and an anaphase bridge is formed, with a high probability of breaking at some point, generating chromosomes with deletion or translocation. In any type of segregation, the acentric fragment can form a micronucleus. (2) Segregation of a tetraradial with two DNA ends rejoining segments without centromere. (2a) Both chromatids with a translocated centromere segregate to the same pole, the result is one daughter cell with balanced translocation and one normal daughter cell. (2b) The translocated chromosomes segregate to different daughter cells, both will have unbalanced translocations. (**C**) Cells from a FA patient showing: (i) anaphase bridge; (ii) interphase bridge, resulting of the segregation failure of a dicentric; and (iii) micronucleus, frequently formed by an acentric fragment that could not join the mitotic spindle.

Translocations, dicentric chromosomes and chromosome deletions may be directly originated during the abnormal processing of ICLs or arise as a consequence of the extremely abnormal segregation that radial figures undergo during mitosis. Depending on the type of radial chromosome, the transition through anaphase will result in the segregation to the daughter cells of translocated chromosomes, dicentric chromosomes, acentric fragments and deleted chromosomes (Figure 4). Importantly, large numbers of cells can succumb to cell death by the accumulation of gross genomic imbalances, i.e., radial figures can lead to anaphase bridges and mitosis blockage, or chromosome fragments can lead to micronuclei formation after cytokinesis, which can create a vicious circle of CIN that can eventually result in the emergence of neoplastic clones. Of note, each cell with at least one radial figure can give rise to four different daughter cells, carrying different non-clonal chromosomal alterations. This makes clear that a cell with several SCA will generate daughter cells with karyotypes different from the progenitor cell, generating a wide diversity of genotypes (Figure 4).

#### 2.3.3. Other Chromosome Aberrations

All of these SCA can be accompanied by numerical alterations, such as aneuploidies (gains or losses of whole chromosomes) and polyploidization. In metaphase spreads of patients with FA, it is relatively common to find tetraploid cells and mitotic figures with endorreduplicated chromosomes, with four instead of two chromatids. FA cells are also known to have alterations in the duration of the cell cycle phases (explained below) or in the transition from one phase into another. These might provoke new DNA replication cycles in the absence of mitosis and cytokinesis, leading to endorreduplicated chromosomes in the next mitosis (Figure 3a) [47]

### 2.4. Chromosome Aberrations for the Diagnosis of Fanconi anemia

**Figure 5:**
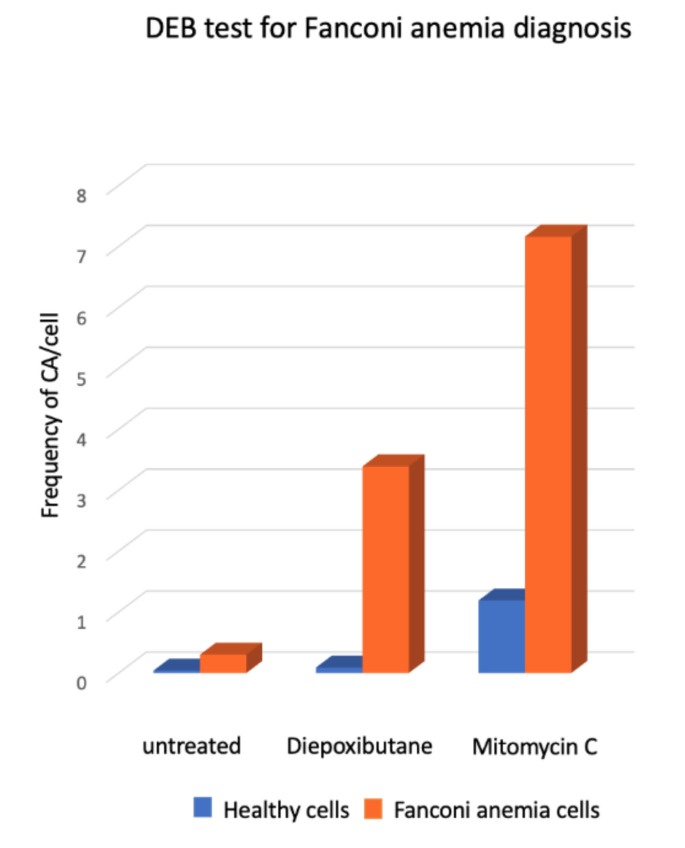
Response of lymphocytes from FA patients (*n* = 18) and healthy subjects (*n* = 117) to treatment with ICL inducing agents, Diepoxybutane [0.1 µg/mL] and mitomycin C [40 ng/mL]. Gross chromosomal aberrations such as radial figures, translocations, deletions and duplications were commonly observed in FA cells [49] Although the two challenge agents are effective, the use of DEB for diagnosis is preferred, because the results are less variable and the difference between non-FA vs. FA cells is generally clearer.

Presence of SCAs is a hallmark of the FA cellular phenotype, therefore the analysis of the number and type of SCA is used in the diagnosis of FA. An approximate 10-fold increase in the DEB-induced frequency of SCA and the presence of radial figures are indicative of FA (Figure 5). In some patients, the diagnostic chromosome breakage test for ruling out FA might turn out to be inconclusive due to the presence of a subpopulation of cells that are not sensitive to DEB or MMC and behave as normal cells. In these cases, the presence of a revertant cell line giving rise to mosaicism should be sought. Mosaicism in the context of FA refers to the existence, in a single patient, of two hematopoietic cell populations, one sensitive and one resistant to ICL-inducing agents. Mosaicism appears due to the reversion of one of the original germline PV causing FA. It is calculated to be present in up to 20% of patients with FA and can have multiple origins, including gene conversion, back mutation, second-site mutation, among others. There is no standard methodology for the diagnosis of mosaicism, however a patient is generally considered to have hematopoietic mosaicism when a sub-population of his/her lymphocytes displays DEB resistance, while their fibroblast show DEB sensitivity [48]. The presence of mosaicism has clinical implications, if the reversion occurs early in the primitive hematopoietic stem cells it might lead to increased blood cell counts, improved aplastic anemia, as well as a reduction in the incidence of bone marrow failure and hematologic neoplasia [48].

## 3. Fanconi Anemia Proteins beyond ICL Repair

### 3.1. Fanconi Anemia Proteins Are Involved in Replication Fork Protection

DNA replication in the human chromosomes starts at thousands of replication origins that are “licensed” by the minichromosome maintenance proteins (MCM2-7) before entering S-phase. Not all the licensed replication origins will fire, most of them will remain as dormant origins [50]. The firing of origins of replication is highly regulated, some are early-replicating and others are late-replicating origins, each one forming bidirectional replication forks [51]. Progression of the replication fork can be challenged by insults of endogenous and exogenous origin that can lead to replication stress; this form of stress appears when the replication fork progression is stalled, forming aberrant replication forks characterized by stretches of single strand DNA (ssDNA) due to DNA polymerase stalling, while the replicative helicase continues unwinding the parental DNA. These stretches of ssDNA are covered by the RPA protein, which activates the replication stress response proteins, with ATR being the main responder kinase that will inhibit the cell cycle progression and will suppress the firing of late-replication origins. The persistence of a stalled replication fork compromises the stability of the replisome complex, which will eventually dissociate from the fork, resulting in fork collapse; the latter leads to the formation of replication fork reversal “chicken foot” structures and DSBs, making them important endogenous sources of chromosomal instability [52].

FA proteins play a central role in protecting DNA replication both under normal and replication stress conditions. In this scenario, the functions of the FA proteins are independent from their role in the repair of ICLs and change depending on the severity of the replication stress [21]. FANCD2 and FANCI proteins are involved in regulating origin firing and replication fork stability during normal growth conditions [53]. Under low levels of replication stress, independently of FANCI and the FA core complex, monubiquitinated FANCD2 recruits the BLM complex to stalled replication forks in order to restart them and prevent firing of dormant origins, while FANCI has a role in firing dormant origins to facilitate proper and timely replication [3,51,54]. The response of FA proteins to replication stress is mediated by ATR; in one scenario, ATR phosphorylates FANCI, promoting its binding to FANCD2 and then suppressing origin firing, while, in the other scenario, FANCD2 is activated by monoubiquitination, and is then targeted to stalled replication forks, where it interacts with the MCM proteins and BLM to promote replication fork stability and induce the restart of stalled replication forks [21,51].

In conditions of high replication stress, components of the FA/BRCA pathway are activated, particularly RAD51/FANCR binds to the ssDNA exposed in the stalled replication fork and form a nucleofilament that is protected by BRCA1/FANCS and BRCA2/FANCD1; in addition, the nascent DNA is protected by FANCC, FANCJ, FANCM and the FA core complex [9,21]. Monoubiquitinated FANCD2/FANCI has recently been shown to have the capability to bind double stranded DNA (dsDNA) and form a nucleofilament that may strongly clamp on reversed replication forks, preventing the access of endonucleases, such as MRE11/DNA2, which can degrade the nascent DNA and cause DSBs [55].

Recently, it has been found that FANCM regulates the correct repair of stalled fork, selecting the HR pathway, which protects fork-remodeling and elicits error-free repair, instead of alternative error-prone repair pathways. Specifically, FANCM, aided by BRCA1/FANCS, BRCA2/FANCD1 and RAD51/FANCR, is able to select the conservative repair mechanism that generates “short-tract” gene conversion and suppresses error-prone “long-tract” gene conversion. On the other hand, FANCM-BLM and BRCA1/FANCS (independently of BRCA2/FANCD1 and RAD51/FANCR) suppress the formation of tandem duplications arising by error-prone replicative responses, thus highlighting the function of FANCM as a key mediator of repair pathway choice at stalled replication forks, to preserve the genomic stability [56].

Collectively, all this information evidences an important role for the FA proteins in protecting DNA replication, beyond ICL repair. Table 3 summarizes the sources of replicative stress (endogenous and exogenous) in which the participation of FA proteins has been demonstrated. General mechanisms that induce replication stress include nucleotide pool depletion, transcription–replication collisions, unusual DNA structures and disturbed origin firing [53].

#### 3.1.1. Nucleotide Depletion

Nucleotides are essential components for DNA replication and may affect FA cells [57]; depletion of the pool of nucleotides by hydroxyurea (HU) increases the frequency of collapsed replication forks. In the presence of HU, the FA/BRCA pathway is activated. In this scenario, FANCD2 protects the nascent DNA from MRE11-mediated degradation, whereas RAD51/FANCR, BRCA2/FANCD1 and BRCA1/FANCS proteins assist in the re-start of stalled replication forks [55].

#### 3.1.2. Transcription-Replication Collision

Collision of the replication and transcription machineries has also been associated with replication stress. During transcription, a DNA:RNA hybrid, known as R-loops, can be formed between the template DNA and nascent RNA. R-loops can interfere with replication fork progression and induce replication fork stalling and collapse. R-loops usually occur at sites encoding large genes of more than 800 Kb or can also be induced by the aldehydes generated during normal cellular metabolism [54]. FA/BRCA pathway is directly involved in the removal of R-loops and in preventing the accumulation of R loop-mediated DNA damage [58]. Excessive R-loops formation can also occur in sites of activated oncogenes with increased transcription [53], in telomeric regions of cells with an active alternative lengthening of telomere (ALT) pathway, or accumulate in the telomeres, where the TERRA RNA (a long non-coding RNA transcribed from the telomere/subtelomere regions) can loop with the DNA molecule. The translocase/helicase activity of FANCM, BLM and other FA proteins, disrupts the TERRA R-loops during telomere replication, suppressing the replicative stress in these regions [59].

#### 3.1.3. Repetitive DNA Sequences

Chromosomal regions with highly repetitive sequences, such as centromeres, telomeres and CG-rich DNA, are difficult to replicate and susceptible to present replicative stress due to the formation of complex DNA secondary structures such as stem-loops, G-quadruplexes (G4) structures and DNA catenates. All of these structures have the potential to interfere with the progression of the replication fork and consequently generate under-replicated DNA regions, formation of UFBs, chromosome breakage and impaired cytokinesis. A functional FA/BRCA pathway is fundamental to prevent replication stress and maintain genomic stability [53,60].

#### 3.1.4. Common Fragile Sites

Common Fragile Sites (CFS) are late-replicating repetitive sequences that may contain tumor suppressor genes or proto-oncogenes. These defining characteristics make them genetically unstable chromosomal regions that are highly susceptible to replication stress and make them hotspots for structural chromosomal aberrations, notably due to: (a) DNA repetitive sequences prone to form secondary structures; (b) transcription–replication collisions (mainly in the presence of active oncogenes); and (c) scarcity of replication initiation (Table 3) [61]. Moreover, CFS are evidenced when HU and aphidicolin (an inhibitor of replicative polymerases) are used and induce under-replicated DNA. The need of a functioning FA/BRCA pathway to preserve the integrity at CFS is made evident in FA patients, in whom over 80% of CA breakpoints are found in CFS [62].

In the absence of exogenous stress, the FA pathway and specifically FANCD2 and FANCI are involved in the protection of CFS [21,61]. Importantly, under-replicated CFS will give rise to ultra-fine bridges (UFB) between sister chromatids that will prevent proper chromatid separation during mitosis. FANCD2 and FANCI will localize to these UFBs and recruit BLM for their processing. Unresolved UFBs in cells with a deficient FA/BRCA pathway lead to cytokinesis failure resulting in chromosome breakage, formation of binucleated cells, polyploidy and apoptosis [21,60,61].

## 4. The Control of the Cell Cycle Checkpoints in FA Cells

As mentioned in the previous sections, the DNA repair deficiency that characterizes FA leads to accumulation of unrepaired DNA damage with cellular and physiological consequences [64]. When facing DNA damage, every cell has to make the decision whether to divide or not based on the amount of DNA damage that the cell is harboring.

**Figure 6:**
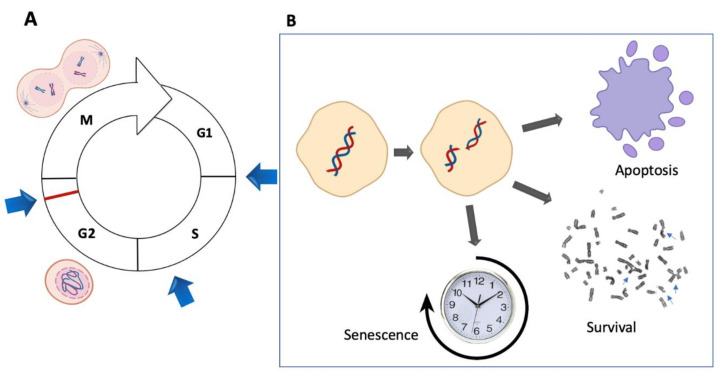
FA cells take cell fate decisions in their transitions through the cell cycle. (**A**) The G1 checkpoint verifies that the cell has the requirements for starting DNA replication, the S phase checkpoint verifies the accurate and timely replication of DNA, the G2 checkpoint verifies that all the chromosomes are correctly replicated and without DNA damage and the M phase checkpoint verifies that chromosomes are correctly aligned to the mitotic spindle before chromosome segregation. Blue arrows indicate moments of interphase cell cycle checkpoints. (**B**) The cell cycle checkpoints are safeguarding moments in which cell fate decisions are taken. For DNA repair deficient cells, such as FA cells, these points are critical since the decision has to be taken whether to activate apoptosis, divide with unrepaired DNA damage or enter into the senescence program.

The normal and timely progression through the cell cycle is controlled by several cell cycle checkpoints functioning during the G1, S and G2 phases [26,65]. These checkpoints monitor the integrity of the DNA molecule, whereas an additional M-phase checkpoint monitors for the appropriate chromosome alignment before chromosome segregation (Figure 6A) [66]. A defect in the G1 phase has been described in FA hematopoietic stem and progenitor cells (HSPCs) [67], but no defects have been described in other tissues or cell lines derived from patients. However, early work suggested an impaired S-phase checkpoint in FA cells that would allow an accelerated S-phase completion at the expense of DNA damage accumulation [68] and an exacerbated G2 checkpoint, which would be used by FA cells to gain time to repair the DNA damage that was allowed to go into the S-phase. This phenotype is easily identified when FA cells are exposed to increasing concentrations of ICL-inducing agents: an almost extinct S-phase and a prominent G2 peak.

The prominent cell cycle arrest to which FA cells are subjected has been ascribed to p53, a cell cycle master regulator [67]. p53 is a transcriptional factor whose better-known function is to fine-tune the expression of genes that control cell cycle arrest and those that regulate apoptosis [69]. p53 can undergo post-translational modifications, mainly phosphorylation, which shape its affinity for certain domains in the promoters of its target genes [70]. This protein can sense the amount of DNA damage and it has even been suggested that the available amount of p53 and its target affinity responds to the amount of DNA damage that a cell has at a particular time. It has been assumed that limited DNA damage leads to the transient activation of p53 and cell cycle arrest mediators, whereas large amounts of DNA damage result in p53 stabilization and activation of pro-apoptotic targets, thus conducting to cell demise [71,72].

Additional mechanisms that allow the escape of FA cells from the strong cell cycle checkpoints are starting to be elucidated. These include the checkpoint recovery, a whole system of phosphatases, led by PPM1D/WIP1, that dephosphorylates ATM, CHK1, p53 and the histone **γ**H2AX to signal the end of the DNA damage response [64]. When this cascade is dephosphorylated, the cell can ignore the DNA damage and divide despite the presence of broken chromosomes. Of note, when bulk FA samples are studied, overexpression of both checkpoint and checkpoint recovery genes can be observed [73]. However, recent single cell RNA sequencing studies have the potential to deconvolute the heterogeneous DNA damage response of FA cells, elucidating whether cells are either arrested or poised for cell division despite the detection of DNA damage (Figure 6B).

## 5. Clinical Consequences of FA/BRCA Pathway Failure

Three main clinical features of FA have long been recognized: (1) developmental alterations; (2) bone marrow failure; and (3) an increased risk to develop cancer. The clinical presentation among patients is highly heterogeneous: not all patients develop all features and there is important variability in the severity of each documented feature.

The complete FA pathway is only present in mammals, but can be found in a reduced version in other organisms [74] The effects of an altered FA/BRCA pathway are not universal within mammals. Although a clear phenotype is recognized in humans, mice models do not recapitulate the complete human FA phenotype [75] These species differences have proven to be an obstacle to model several FA features, and mechanistic studies that explain phenotypic outcomes are scarce.

### 5.1. Development Alterations

A recent literature review of published cases, which analyzes the reported physical features of the largest number of confirmed patients with FA, found that almost 80% of them had at least one physical feature, the more frequent ones were: short stature, upper limb radial ray abnormalities, skin pigmentation changes, renal malformations and central nervous system findings [8].

Morphogenesis is a highly regulated process, there are critical moments during development where different organs can be particularly sensitive to insults [76].

Aldehydes are also a byproduct of alcohol metabolism, alcohol has been shown to rearrange chromosomes and kill cells [77] and its teratogenic effects are clearly shown in children born to mothers who have ingested alcohol during pregnancy manifesting as fetal alcohol spectrum disorders (FASD), where developmental issues and malformations are important features.

It could be speculated that the hardship found by patients with FA during development to take care of the ICL resulting from endogenous acetaldehydes could contribute to the physical phenotype of patients with FA. It has been hypothesized that the overlapping features in FA and FASD are the consequence of aldehyde susceptibility of somatic stem and progenitor cell populations [78]. ALDH2 genotype has been proposed to be a phenotype modifier in FA, the A allele has been found to be associated to early bone marrow failure progression as well as an extensive malformation phenotype [79]. Moreover, a more severe phenotype has been observed in individuals with an ALDH2-AA genotype from three different sibling pairs with FA (same *FANC* gene genotype and similar genetic background among siblings) [80].

Anthropometric features are key components of the classical FA physical phenotype. Short stature is reported in half of the patients and low birth weight is also a commonly reported feature of patients with FA [81]. Evidence in mice support the hypothesis that growth retardation and short stature in humans with FA could be due to the loss of pluripotent stem cells during embryogenesis [82]. FA mice (mutant for *Fancd1* and *Fancn*) die early during embryonic development due to increased apoptosis [82]. Nevertheless, short stature has not been found to be associated to patients with PV in *FANCD1* and *FANCN* genes, but has been linked to genotypes of downstream genes [8]. The association to genes from the downstream part of the FA/BRCA pathway has also been found for a small head; microcephaly, described in almost 30% of published cases [8], may reflect the importance of appropriate DNA repair in neural progenitors undergoing rapid replication cycles during central nervous system development [83,84].

Upper limb radial ray abnormalities are also a pivotal feature that brings the FA diagnosis into the minds of clinicians. It has been estimated that up to 1% of patients with congenital thumb malformations have FA [85]. The upper limb phenotype in patients with FA is extremely variable: most patients have normal structures, but, for the 40% who have a radial ray abnormality [8], the severity spectrum is vast as it can go from discrete flat thenar eminences to obvious oligodactyly or polydactyly [86], and it may affect either a single or both upper limbs [87]. The pathophysiologic basis of radial ray abnormalities in FA remains unknown [85], yet it is well recognized that genetic factors have an important role in the pathogenesis of radial ray deficiencies, for instance fibroblast growth factor (FGF) expression has been identified as necessary for appropriate radial development [88]. Although this has not been explored in the context of FA, it is possible that the ubiquitous FA/BRCA pathway may somehow interact with other developmental pathways adding to the stochastic factors contributing to the variability of upper limb phenotypes in patients with FA [41].

The relevance of stochastic factors in developmental phenotypes is also illustrated by renal malformations. The patterns of abnormalities found in these paired organs point to disruption of migration patterns of embryonal organs to their final position occurring at an early developmental stage, suggesting that the FA pathway may have a role in this [89]. A literature review estimates a frequency of kidney malformations in nearly 30% of patients [8], but this seems to be an understatement since intentional assessment of renal anatomy has shown that nearly 50% of studied patients have alterations.

**Figure 7:**
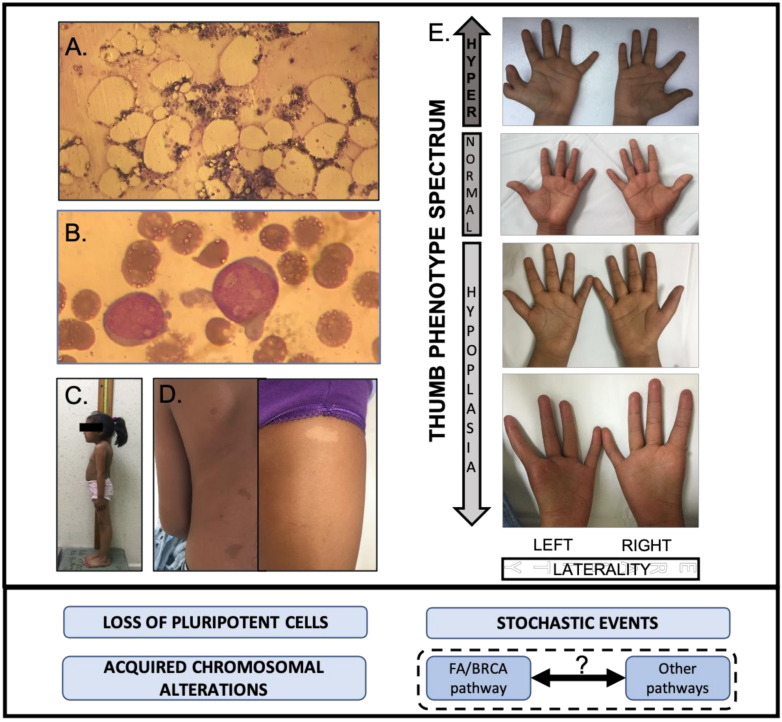
Deficit of the FA/BRCA pathway impacts the Fanconi anemia phenotype. (**A**) Bone marrow failure results from bone marrow attrition due to loss of hematopoietic progenitors. (**B**) Acute myeloblastic leukemia frequently has acquired chromosomal aberrations. (**C**) Somatometric features: short stature and microcephaly are thought to express an early loss of pluripotent cells during development. (**D**) Pigmentation: A hypothesis is that café au lait and hypochromic macules could be subjacent to acquired chromosomal alterations in skin cells. (**E**) Thumb abnormality spectrum. Stochastic events are credited for the high variability found in malformative phenotypes of patients with FA, while the interaction of the FA/BRCA pathway with other developmental pathways remains to be investigated.

A more flagrant misestimation of occurrence is found for skin pigmentation changes, which have been described in almost 40% of published cases of patients with FA [8]. However, a recent study designed to delineate the cutaneous findings in FA found, after direct examination, that almost all patients with FA had at least one pigmentary alteration. The more frequent being café au lait macules, but also identifying hypopigmented macules of which the skin-fold freckle-like macules variety could be characteristic of FA [90]. It has long been established that anomalous pigmentation is associated to chromosomal alterations, and that they may only be found when skin tissue is analyzed [91,92]. Pigmentary changes in FA have not been thoroughly studied and are poorly understood, although an evident hypothesis is that they respond to accumulated genomic instability. The fact that pigmentary changes in FA appear to increase with age supports this possibility and that they occur in both exposed and non-exposed areas [90] would support that the CIN does not result from UVA exposure but other types of damage, such as the one arising from a defective FA/BRCA pathway (Figure 7).

### 5.2. Hematological Manifestations

Bone marrow failure (BMF) is the more characteristic feature of patients with FA. It has been estimated that the cumulative incidence of severe BMF reaches 70% by age 50 years, with a median age at presentation of seven years [93,94].

Multiple reports suggest a direct role for p53 hyperactivation as an important mechanism of BMF in FA patients [67,95,96,97]. The primary genetic defect of FANC genes present in hematopoietic stem cells (HSC) hinders their ability to deal with replicative stress during prenatal HSC expansion by triggering an apoptotic p53/p21 mediated response that results in a prenatally reduced fraction of CD34+ cells [67]. This compromised HSC pool is further challenged by DNA damage accumulation during extrauterine life. Reactive aldehydes, byproducts of normal cellular metabolism, are important genotoxins neutralized by the FA/BRCA pathway; the exposure of FA deficient cells to aldehydes results in the accumulation of chromosomal aberrations [98]. Aldehyde dehydrogenases enzymes are known to be important for aldehyde detoxification [99]. Mice HSC have been found to heavily rely on the Aldh2 enzyme function to protect from aldehyde toxicity; double Fancd2-/- Aldh2-/- mutants have a severe defect in their HSC pool that correlates with an increase in the DNA damage marker **γ**H2AX [100]. It is widely accepted that BMF in patients with FA is driven by endogenous-aldehyde induced toxicity of HSC cells, although cytokine overproduction has also been hypothesized to contribute to the BMF phenotype in FA, but it is not yet clear if this sensitivity is related to the DNA repair deficiency or results from alternate roles of FA proteins [101].

FA cells also overexpress other cell cycle regulators such as the ATM and CHK1 kinases which are important checkpoint overseers. Both these kinases have the capacity to phosphorylate p53. Moreover, ATM is also capable of phosphorylating CHK1 (although its canonical target is considered to be CHK2) in response to DNA damage [102,103]. The effect of this is the induction of a strong cell cycle arrest in basal conditions of FA cells. Of note, an attenuation in the characteristic cell cycle arrest of FA cells has been observed in certain FA patients through the downregulation of CHK1, which however allows the division of cells with unrepaired DNA damage [104].

Until recently, bone marrow failure in FA has been thought as the consequence of excessive growth suppressive pathways that in addition to p53/p21 include the hyperactivation of the potent growth inhibitory TGFβ pathway [105]. However, the mere existence of FA patients suggests that mechanisms allowing their survival must exist and counteract the growth suppressive activities of p53 and TGFβ pathways. Recent data illuminate this through single cell RNA sequencing of primary HSPCs from FA patients that showed overexpression of the MYC oncogene occurs in a subset of FA HSPCs and appears to be a counteracting force against the growth suppressive activities of TGFβ and p53, since inhibition of MYC expression reduces the proliferative capacity of FA HSPCs. MYC overexpression in FA cells, however, is a double-edged sword that allows the progression of FA cells through the cell cycle but at the same time increases their replicative stress [106] and concomitant CIN, a FA characteristic that is tightly related to clonal evolution that precedes neoplasia.

### 5.3. Oncologic Susceptibility

Patients with FA have a significant risk to develop cancer. The cumulative incidence of leukemia has been estimated to be under 5% by age 30, while the myelodysplatic syndrome (MDS) cumulative incidence was found to be 50% by age 50. Regarding solid tumors, the cumulative incidence is about 20% by age 65, with a hazard rate that increases exponentially after the age of 30. This translates into a reduced median overall survival of patients with FA of 39 years [93].

#### 5.3.1. Hematologic Neoplasias

##### MDS

Non-transplanted patients with FA have an outstanding risk to develop MDS, which has been shown to be over 5500-fold that of the general population. The hazard rate of MDS reaches 1% by age 10 [107], and the cumulative incidence reaches 50% by age 50 [93]. The most frequent MDS subtype found in patients with FA is refractory cytopenia with multilineage dysplasia. This morphologic diagnosis is usually reached when the bone marrow shows dyserythropoiesis, a feature found in over 90% of patients, accompanied by over 10% dysplastic cells in one or two other myeloid cell lines [108]. Morphologic data of MDS are correlated to clonal evolution [108]; the MDS progression to leukemia has been estimated at 9% in patients with FA [109].

##### Leukemia

Leukemia has been found to occur in 3% of patients with FA [93]. In over 80% of the cases, it is acute myelogenous leukemia (AML) [107], although cases of acute lymphoblastic leukemia (ALL) have also been reported [110]. Hazard for AML rises steadily after age 10 and plateaus by the age of 20–30 years [107] so that by age 30 cumulative incidence of leukemia has been estimated to be under 5% [111]. To our knowledge, AML FAB subtypes in patients with FA have not been reported [112]. However, it is well known that patients with FA do not have recurring rearrangements such as t(8:21); inv (16) and other aberrations usually found in de novo AML and that aid in classifying according to FAB subtypes [113]. The signature recurring aberrations found in the bone marrow of FA patients bear witness to clonal evolution mediated by deficiency of the FA/BRCA pathway.

##### Bone Marrow Abnormalities

Up to 40% of children and young adults with FA exhibit signs of clonal evolution in the bone marrow [114], while up to 15–60% of patients, depending on the cohort, may develop MDS/AML [94,111,115]. Clones can be a frequent finding in the bone marrow aspirates of FA patients, even before having any morphological sign of MDS or AML progression [116]. However, additional chromosomal abnormalities below the microscopic detection limit might add to the actual frequency of clones.

Progression to MDS and AML in FA is associated with the presence of clonal and non-clonal chromosomal alteration, and both are valuable biomarkers for detecting progression to cancer. This agrees with the theory of CIN as a driver of the evolution to cancer. In fact, as shown in Figure 4, in FA cells, CIN constantly generates cells with different karyotypes due to non-clonal chromosomal alterations. These gross changes in the karyotype are important, since each cell has a specific genome reorganization that generates a new genome system [117].

Cytogenetic and next generation sequencing analysis of MDS and AML bone marrow samples from patients with FA have identified gross chromosomal abnormalities. The most frequent findings include partial duplication of chromosome 1q (1q+, 44.8%), partial duplication of chromosome 3q+ (41.3%), duplications in 21q+ (20.7%), monosomy of chromosome 7 or deletion of chromosome 7q− (17.2%) and 11q+ (13.8%), whereas mutations are more commonly in the genes *RUNX1* and *RAS* [116,118].

Although some of the chromosome abnormalities mentioned above are shared between patients with FA and MDS/AML from the general population, mutations in MDS/AML oncogenes and tumor-suppressor genes classically found in MDS/AML samples are rarely found in FA. On the other hand, chromosomal lesions that seem to be specific to FA include 1q+ and 3q+; of note, 1q+ has been observed in the BM of patients with FA in all MDS/AML stages and even in normocellular bone marrow or hypoplastic bone marrow without signs of transformation, suggesting that 1q+ clones might confer a survival advantage to the HSPCs from FA patients without being a part of the malignant transformation process [116,118].

#### 5.3.2. Solid Tumors

Solid tumors were found in 12% of patients with FA. Major cancer sites have consistently been reported as head and neck squamous cell carcinoma (HNSCC), vulva, esophagus and brain. Their cumulative incidence is about 20% by age 65, with a hazard rate that increases exponentially after the age of 30. This translates into a reduced median overall survival of patients with FA of 39 years. Overall, a 19 observed/expected ratio for all solid tumors in non-transplanted patients with FA was found, with a median age of presentation of 34 years. An increased risk of solid tumors in transplanted patients was confirmed [93]. Molecular analysis of a subset of squamous cell carcinomas (SCC) from patients with FA showed that the allelic loss in these tumors is similar to sporadic SCC, suggesting that the same genes and chromosomal locations are targeted in SCC irrespective of their etiologic cause [119]. Genomic instability in epithelial cells from patients with FA has been evidenced by two recent studies. The first one evaluated the frequency of micronuclei (MN) in exfoliated buccal cells from patients with FA, in whom a higher frequency of MN was found when compared to a control group, and an even higher frequency was observed in patients who had had SCC. The MN frequency was found to be such a good biomarker of chromosomal fragility in epithelial cells that it is already being studied as an endpoint in clinical trials for chemoprevention interventions [120]. The second one studied brush biopsy specimens from oral lesions of patients with FA and showed that DNA aneuploidy is a good biomarker for oral epithelial dysplasia or SCC [121].

#### 5.3.3. Skin Cancer

Skin cancer is also extremely frequent in patients with FA. In the NCI cohort, the overall number of non-melanoma skin cancers in both non-transplanted and transplanted patients is larger than the number of solid tumors, and over 70% (8/11) of patients who had skin cancers had more than one [93]. The published information concerning skin cancer in patients with FA is scarce and vague; more precise data gathering could shed light into factors that may contribute to their development, such as the anatomical region where they develop and if they initiate in skin with pigmentation changes. An interesting observation made by Kao et al. when analyzing DNA repair pathways in different skin cancers is that the FA/BRCA pathway may be contributing to melanomagenesis, since genes from this pathway were found to be upregulated in melanoma tumors [122]. It is interesting that melanoma was not reported in the NCI cohort and, to the best of our knowledge, has not been reported in any patient with FA. A hypothesis is that the deficiency in the FA/BRCA pathway may be protecting patients with FA from this kind of cancer.

#### 5.3.4. Childhood Solid Cancer

Besides the more frequent solid tumors such as HNSCC, there are around 40 reported cases of FA patients in whom primary solid tumors have been found during childhood. The known genotypes of these patients are either *FANCD1 (BRCA2*) or *FANCN (PALB2*), in whom cancer usually occurs in the first decade of life [123,124]. The type of tumors associated with this presentation are brain tumors, nephroblastomas and neuroblastomas, and many patients with such genotypes develop multiple primary neoplasms. Microarray-based CGH analysis of tumors from these patients has shown a large number of segmental alterations, but they do not show a recurrent pattern, as has been described for MDS and leukemia in patients with FA. However, the alterations found have been associated to aggressive phenotypes [123].

#### 5.3.5. Increased Risk for Heterozygotes

Most patients with FA bear mutations in autosomal genes that require biallelic mutations to reveal the recessive character of the disease. In a classic paper from 2002, Howlett et al. linked, in the same pathway, known *FANC* genes and the hereditary breast and ovarian cancer (HBOC) genes *BRCA1* and *BRCA2* [125], substantiating the observation that some family members from patients with FA could have an increased risk of cancer. Family members of patients with FA who are heterozygous carriers of PV in genes from the fourth module *FANCD1 (BRCA2), FANCJ (BRIP1), FANCN (PALB2), FANCO (RAD51C)* and *FANCS (BRCA1*) (Figure 1) are at increased risk to develop cancer [2,21]. As a corollary, carriers of PV in those genes are at increased risk of conceiving children with FA if their partners are also carriers [43].

#### 5.3.6. Somatic Mutations in *FANC* Genes in Sporadic Cancer

The ability to perform DNA repair is a central mechanism for the protection of genome stability. It is of no surprise then that inappropriate genome maintenance conducts to cancer. Genes from the FA/BRCA pathway are of course susceptible to acquiring mutations in the course of malignant transformation for a number of cancers. The sporadic forms of cancers typically found in patients with FA (breast, ovarian, HNSCC cancers and with a lesser frequency AML) have been reported to have an assortment of somatic mutations in genes from the FA/BRCA pathway. This somatic susceptibility for mutations in genes of the FA/BRCA pathway is not limited to such cancers, but can also be found in infrequent cancers in FA such as melanoma, evidencing the importance of this pathway for genomic stability in a myriad of cell types [43].

### 5.4. Infertility

The lack of a functional FA/BRCA pathway can have important and adverse consequences in the gonadal function, and not surprisingly fertility issues have been reported in both female and male patients with FA. On the one hand, ICL mis-repair would lead to gross chromosomal rearrangements that will affect the mitotic divisions that germ cells undergo before entering meiosis (millions of cells in the male germ line), leading to death of cells with chromosome breaks and genomic imbalances that provoke oligospermia or azoospermia in male FA patients. On the other hand, if cells with balanced chromosome rearrangements (translocations or inversions) move into the prophase I of meiosis, the pairing that the homologous chromosomes undergo during zygotene might be impaired, thus preventing the synaptic events needed for recombination of the homologous chromosomes or generating complex meiotic figures during pachytene, which subsequently could stop the meiotic division and trigger apoptosis [47]. The third process that might impair gonadal function in FA patients is the involvement of FANC proteins in the appropriate progression of meiosis, both during the process of programmed DSBs and during meiotic recombination [48]

Due to gonadal dysfunction, pregnancies are rare events in FA; to our knowledge, there are fewer than 50 pregnancies reported in the literature. Although both transplanted and non-transplanted patients have become spontaneously pregnant [78], their pregnancy rates are under 15% [126]. Most of these pregnancies occurred when the women were in their early twenties, which is not surprising since premature ovarian insufficiency (POI) has been found to be a feature of FA [126]. Moreover, heterozygous rare variants in *FANC* genes have been found in patients with non-syndromic POI [127], and male subjects have been identified as having FA after molecular diagnosis was ordered during causal investigation for azoospermia [128], substantiating the role of the FA/BRCA pathway in fertility.

Mice models have proven essential to further understand the mechanisms that result in subfertility in FA. The FA/BRCA pathway is needed in the response to replication stress; it becomes essential in situations of rapid proliferation such as expansion and maintenance of primordial germ cells (PGC) [129]. Mice studies have shown that non-functional Fanc proteins result in PGC attrition [129,130,131,132]. Although the FA/BRCA pathway is essential for ICL repair in somatic cells, the core complex does not seem essential for programmed DSB during meiosis, yet the role of FA proteins in mammalian meiosis has not been largely studied [133]. Studies in diverse non-mammalian species have shown that FA proteins have a role in meiotic recombination during DSB repair and crossing-over, but the attrition PGC phenotype seen in mammals is probably masking the meiotic phenotype in mice [133].

## 6. The Dichotomy of Aging and Cancer in FA

Many of the features discussed in the previous section can also be found in individuals from the general population, but they develop at later stages of life when the individuals belong to the elder population [134]. Patients with FA typically develop aplastic anemia, myelodysplastic syndrome, acute myeloid leukemia [110,135] and premature ovarian insufficiency [126,133] at an early age, although not treated here they also have been reported to have osteopenia/osteoporosis and diabetes [136] when young. A phenotype that combines all of these features gives a place to FA among the premature aging disorders [134].

**Figure 8:**
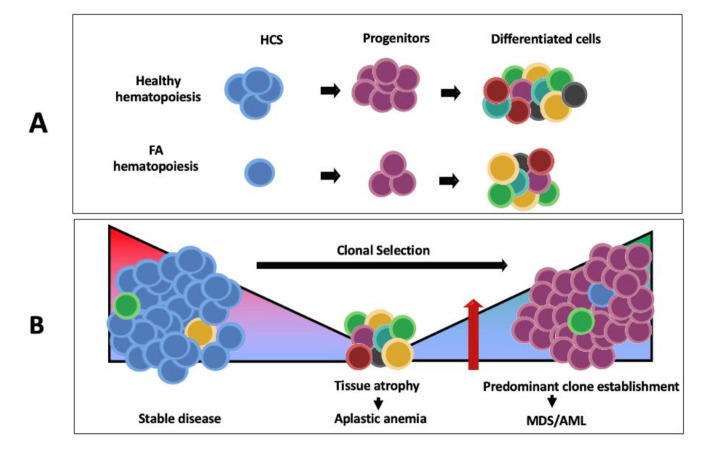
HSPCs in FA are subject to a strong selective pressure. (**A**) The reduced number of hematopoietic stem cells in the bone marrow of patients with FA leads to reduced numbers of progenitor cells and differentiated cells. (**B**) Young patients with FA usually have a more stable disease, however DNA damage and environmental stressors might lead to a dramatic reduction in the number of HSPCs. This sweeping of fragile HSPCs might potentially select apoptosis-resistant hematopoietic clones with acquired somatic mutations that eventually could give rise to premalignant hematopoiesis, such as MDS or AML.

Aging in the tissues from FA patients is associated to ineffective cell divisions, especially in the HSPCs compartment, leading to impaired supply of new functional cells and tissue attrition (Figure 8A); however, division of cells with unrepaired DNA damage can still occur, enticing the appearance of malignant/premalignant clones that can develop into cancer and out-take the tissue (Figure 8B) [134]. Importantly, aging and cancer are considered opposing processes. On the one hand, aging develops over the lifespan of a tissue and results from accumulation of detrimental mutations that impair the correct execution of cellular functions. Aged tissues are characterized by accumulation of senescent cells and increased apoptotic rates. On the other hand, cancer results from accumulation of mutations that confer a survival and proliferative advantage, and, with unrestrained cell division capacity, these cells can generate a tumor.

Paradoxically, although cancer and aging are considered antagonists, age is the most significant risk factor for cancer development with the majority of cancers being diagnosed after the age of 65. However, in patients with FA, cancers typically appear at a remarkable young age [94,109,111]. This dichotomy between aging and cancer stresses the relevance of a functional FA pathway, which becomes situated at the crossroads between appropriate tissue maintenance and cancer.

The hallmarks of aging are grouped into three main categories: (1) primary hallmarks, considered to be the origin of cellular damage; (2) antagonistic hallmarks, considered to be compensatory or antagonistic responses to the damage, they initially mitigate the damage but might eventually become deleterious themselves; and (3) integrative hallmarks, responsible for the functional tissue decline associated with aging [137]. At the cellular level, FA cells meet several hallmarks of aging, some of them have been very well characterized in FA, whereas some others, although potentially present in FA, have remained understudied.

Primary hallmarks of aging include genomic instability, telomere attrition, epigenetic alterations and loss of proteostasis (the mechanisms that maintain correctly folded proteins and correct protein turnover) [137]. For all of these, genomic instability is the most prominent primary hallmark of aging in FA [134], whereas telomere shortening has been shown to be subtle [138] and epigenetic alterations and defective proteostasis remain poorly studied in FA.

Antagonistic hallmarks of aging include deregulated nutrient sensing, mitochondrial dysfunction and cellular senescence [137]. Mitochondrial dysfunction has been described in FA and is gaining relevance [139], whereas cellular senescence in FA remains a matter of debate [70,140].

Finally, integrative hallmarks of aging, considered the culprits of the phenotype, are the exhaustion of tissue specific stem cells and altered intercellular communication [137]. Exhaustion of the stem cell pool becomes evident in FA as the dramatic decline of HSPCs takes place at young ages [67], whereas defective intercellular communication involves changes in communication between tissues. An example of aging-associated alteration in intercellular communication is inflammation [137,141]. Acute inflammation events are commonly triggered by pathogen infections, excessive DNA damage (for example during chemotherapy), UV radiation and physical trauma [141]. Another type of inflammation, known as sterile inflammation, is considered to be low-grade and chronic, independent of pathogen infection, specifically associated with aging and also known as “inflammaging” [141,142].

Acute inflammation is a transient response to infection or tissue damage that is beneficial and facilitates tissue repair; however, sterile inflammation is a chronic sustained process, probably promoted by incomplete resolution of the initial stimuli and might ultimately result in tissue remodeling and dysfunction. Sterile inflammation is thought to result from exposure to various endogenous and environmental insults throughout the entire lifespan of a person [137,141,142].

Several agents can activate sterile inflammation including debris from macromolecules, microbial components or extracellular and cytoplasmic DNA fragments, collectively known as damage-associated molecular patterns (DAMPs). DAMPs can activate innate immune cells (neutrophils, macrophages and dendritic cells) and non-immune cells (epithelial cells, endothelial cells and fibroblasts) through the transmembrane pattern-recognition receptors of the Toll-like receptor (TLR) family. TLRs in turn activate the NF-kB transcription factor that upregulates various pro-inflammatory cytokines, including TNFα, IL-1β, IL-12 and Interferons [143].

Although DAMPs-mediated sterile inflammation has not been coined in FA, a pro-inflammatory phenotype has been very well described and includes increased production of pro-inflammatory cytokines, including TNFα and IFNγ, and increased C-Reactive protein (CRP) [144,145,146]. Importantly, the inflammation observed in FA, either acute by infections or sterile, can trigger HSPCs senescence or apoptosis. Both processes are strong tumor suppressors and prevent the damaged FA cells from undergoing division [146,147]. However, if tissue regeneration is not efficient or at an appropriate rate, these two processes can derive into depletion of HSPCs, tissue degeneration and function loss, all of which are aging hallmarks. This lead to hypothesize that recurrent inflammatory events in patients with FA might contract the HSPC pool [114] or that a constant low-grade chronic sterile inflammation, which remains unexplored in FA, might contribute to tissues attrition (Figure 8A).

In FA, however, adaptation of HSPCs to the harsh bone marrow microenvironment can lead to survival and selection of clones resistant to the pro-apoptotic and pro-senescent mechanisms. For example, the inflammatory episodes mentioned above can serve as “selective sweeps” that get rid of non-fitted HPSCs and permit the evolution of clones with the capacity to tolerate the stressors [148,149]. The environmental challenge therefore creates an opportunity for selection and emergence of HSPCs with somatic mutations or epigenetic alterations. In this process, aberrant HSPCs will replicate with more success than their competitors and can give rise to malignant progeny that can overtake the bone marrow (Figure 8B) [150].

This selection can fine-tune, similar to aging, a clonal drift in the composition of HSPCs populations in the FA bone marrow; this drift is typically characterized by a decline in the frequency of lymphoid committed HSPCs and an increase in the frequency of myeloid committed HSPCs [150,151]. Differences in the response and tolerance to DNA damage might operate behind this drift in HSPCs clones, but these differences remain unexplored in FA.

Although the above-mentioned adaptations would extend the survival of HSPCs under hostile microenvironmental conditions, the clonal progeny might acquire subsequent abnormalities in mechanisms controlling growth and differentiation, thus diverting from the original clone and giving rise to MDS and AML.

## 7. Conclusions

The FA/BRCA pathway coordinates the repair of ICLs through the error-free DNA repair mechanism known as HR. When constitutional PV in *FANC* genes render this pathway non-functional, the FA phenotype arises. Clinically, it is characterized by a highly variable presentation including any or a combination of developmental alterations, bone marrow failure and an extremely high risk to develop cancer. In sharp contrast, the cellular phenotype is markedly constant; it is characterized by a prolonged G2 cell cycle phase, proclivity to apoptosis and most notably by CIN. This cellular phenotype is true irrespective of the role performed by the affected FANC protein in the intricated FA/BRCA pathway; it serves as a reminder that FANC proteins function comprehensively to protect the integrity of the genome.

A non-functional FA/BRCA pathway translates at the cellular level into the continuous generation of DSBs, which promote the formation of SCA that are themselves susceptible to further generate other chromosomal alterations, bolstering a vicious cycle of CIN. The resulting load of DNA damage accumulated in FA cells leads to hyperactivation of cell cycle checkpoints that impede cellular division. Unsatisfied checkpoints can concurrently drive increased apoptotic rates or the activation of the senescence program. However, a proportion of FA cells manage to either ignore the cell cycle checkpoints or to override apoptosis giving rise initially to cells with non-clonal chromosomal aberrations that can eventually promote the formation of genomic rearrangements that propel the evolution of malignant clones.

**Figure 9:**
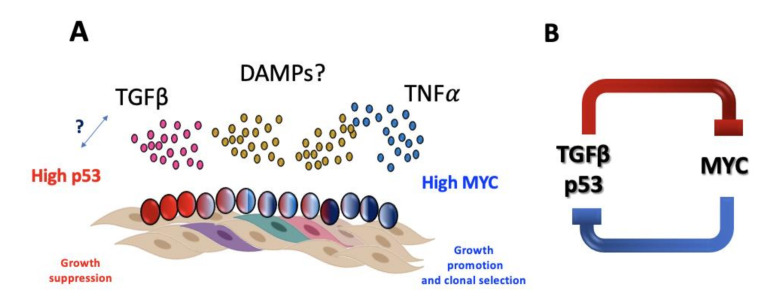
HSPCs in different functional states co-exist in the FA bone marrow and respond differentially to microenvironmental stimuli. (**A**) Overexpression of different pro-inflammatory cytokines has been described in FA and can have opposite effects on responsive HSPCs. On the one hand, TGFβ inhibits the proliferation of FA HSPCs. It is currently unknown if TGFβ and p53 are functionally linked in FA HSPCs, however both proteins suppress proliferation and have increased levels in the FA bone marrow. On the other hand, TNF*α* promotes the proliferation of FA HSPCs, at the expense of DNA damage, by activating the expression of the oncogene *MYC*. TNF*α* is considered a DAMP that promotes sterile inflammation, however the existence of additional DAMPs in the bone marrow of FA patients remains unexplored. (**B**) MYC has opposite transcriptional activities to p53 and TGFβ; its overexpression in FA HSPCs suggests a counteracting force against the growth suppressive activities of TGFβ and p53.

In brief, the two alternatives for cells that cannot repair their damaged DNA are cell death and survival despite genomic damage; these two cellular fates are at the center of the phenotypic presentation of patients with FA. On the one hand, loss of pluripotent stem cells, particularly when subjected to replicative stress during expansion, affects myriad tissues and partly accounts for features such as short stature, microcephaly, infertility and the more extensively studied bone marrow failure, which is currently more thoroughly understood. In the bone marrow, HSPCs with genomic damage can activate the p53 dependent apoptotic program, stop proliferating in a TGFβ dependent manner or divide, mediated by the overexpression of the *MYC* oncogene. *MYC* overexpression is thought to preserve the population of HSPCs that allows the survival of patients with FA (Figure 9B). Nevertheless, most patients with FA eventually develop bone marrow failure, suggesting assistance of additional mechanisms that disrupt this precariously balanced state. Acute inflammation events may contribute to the decline of HSPCs; this may be triggered by excessive DNA damage, infection by diverse pathogens or even sterile inflammation, which is independent of pathogen infection but could well be contributing to the pro-inflammatory phenotype of FA patients. On the other hand, the pro-inflammatory bone marrow microenvironment in FA, partly mediated by TNFα and additional DAMPs (Figure 9A,B), could be sweeping fragile HSPCs out of the marrow while promoting the emergence of hematopoietic malignant clones resistant to apoptosis. Moreover, there is reason to hypothesize that a possible explanation for pigmentary features of FA lies in the development of chromosomal alterations in skin cells.

The lack of animal models that fully recapitulate the human FA phenotype have particularly added difficulty to the study of mechanisms that give rise to the malformative phenotype of FA; today, its pathophysiologic basis remains mainly unknown. Stochastic events, including aldehyde exposure at particular developmental stages, appear to be important; however, mechanistic investigations into the physical phenotype of patients with FA is an area awaiting to be explored.

## Figures and Tables

**Table 1:** Fanconi anemia genes involved in FA/BRCA pathway ^1^.

FANC Gene/Alias	Cytogenetic Location	Function of the FANC Protein
*FANCA*	16q24.3	FA core complex
*FANCB*	Xp22.2	FA core complex
*FANCC*	9q22.32	FA core complex
*FANCD1/BRCA2*	13q13.1	Homologous recombination. Enable RAD51 to displace RPA from ssDNA.
*FANCD2*	3p25.3	Monoubiquitinated ID complex recruits the downstream repair proteins and facilitates repair of DNA ICLs
*FANCE*	6p21.31	FA core complex; bridge between the FA core complex and FANCD2
*FANCF*	11p14.3	FA core complex
*FANCG/XRCC9*	9p13.3	FA core complex
*FANCI*	15q26.1	Monoubiquitinated ID complex recruits the downstream repair proteins and facilitates repair of DNA ICLs
*FANCJ/BRIP1*	17q23.2	FA core complex
*FANCL*	2p16.1	E3 ubiquitin-protein ligase, monoubiquitination of FANCD2
^2^ *FANCM*	14q21.2	FA core complex. Acts by sensing stalled fork by ICLs and recruiting the core complex proteins to the site of ICL
*FANCN/PALB2*	16q12.2	Homologous recombination
^2^ *FANCO/RAD51C*	17q22	Resolution of D-loop structures through Holliday Junction Intermediates and Homologous DNA Pairing and Strand Exchange.
*FANCP/SLX4*	16p13.3	Cooperate with FANCQ-XPF to generate endonucleolytic incisions to unhook the ICL.
*FANCQ/XPF*	16p13.12	DNA endonuclease, involved in homologous recombination; responsible for 5′ incision to remove ICLs
^2^ *FANCR/RAD51*	15q15.1	Interact with the ssDNA-binding protein RPA, RAD52 homologous pairing and strand transfer of DNA
^2^ *FANCS/BRCA1*	17q21.31	Homologous recombination
*FANCT/UBE2T*	1q32.1	E2 ubiquitin-conjugating enzyme, associates with FA core complex, catalyzes monoubiquitination of FANCD2 in association with FANCL
*FANCU/XRCC2*	7q36.1	Homologous recombination
*FANCV/REV7*	1p36.22	Translesion DNA synthesis
*FANCW/RFWD3*	16q23.1	RING-Type E3 Ubiquitin Transferase

^1^ Cytogenetic location was obtained from OMIM. ^2^ Also called Fanconi anemia-like genes, mainly due to the absence of bone marrow failure in the patients [2,9,10].

**Table 2:** Characteristics of the major double-strand break repair mechanisms [22,25,26].

	Non-Homologous End-Joining	Microhomology Mediated End-Joining	Homologous Recombination
Timing	Fast	Fast	Slow
Template dependence	Independent	Independent	Dependent
Homology usage	0–4 bp	2–20 bp	>100 bp
End resection	no	yes	yes
Cell cycle phase	G1, S, G2	G1, S, G2	S/G2
Accuracy of repair	Mostly accurate, error prone	Frequently error prone	Highly accurate

**Table 3:** Participation of the FA/BRCA pathway in the resolution of replicative stress from diverse sources.

Causes of Replication Stress	DNA Lesion/Configuration	Proteins Involved in Replication Stress Resolution ^1^	Outcomes of Unsolved Replication Stress	References
**Exogenous Sources**				
Aphidicolin	Late replication/recombination intermediates	**FANCD2, FANCI,** BLM, PICH	Chromosomal aberrations, UFB in CFS and telomeres, telomere fragility, cytokinesis failure, binucleated cells	[55,60]
Anticancer drugs (Hydroxyurea)	Nucleotide depletion induced reversed forks “chicken foots”	**BRCA2/FANCD1, FANCD2, BRCA1/FANCS, RAD51/FANCR**		
**Endogenous Sources**				
Complex/repetitive DNA sequences:				
Transcription-replication collision	R-Loops	**FANCM, FA pathway, FANCD2**, BLM	Chromosomal aberrations, micronucleus	[53,54,58,59]
Centromere	Catenated DNA Stretched DNA Chromosome entanglements	**FANCM, FANCD2,** BLM, PICH, TOPOIIa, TOPBP1, WRN, TRF1, TRF2	Centromeric UFB, Incomplete chromatid disjunction. Cytokinesis failure, Chromosome breakage, micronuclei, binucleated cells, polyploidy	[3,53]
Telomere	Unresolved R-Loops within ALT telomeres	**FANCM-**BLM, **BRCA2/FANCD1, BRCA1/FANCS, RAD51/FANCR**	Telomeric UFB, Cytokinesis failure, Chromosome breakage	[59,63]
GC-rich DNA	G-quadruplexes, stem loops	**FANCJ**	Chromosome breakage	[53,63]
Common Fragile sites	Large replicons, scarcity of replication origins	MUS81-EME1, ERCC1-**XPF/FANCQ, SLX4/FANCP, FANCD2**, BLM-RMI1-RMI2-TOPOIII;	Fragile sites UFB, Chromosomal aberrations, cytokinesis failure, binucleated cells, chromosome mis-segregation, cell death	[21,55,60,61]

^1^ In bold, FANC proteins involved in replication stress response.
